# Renal Dysfunction and Cardiorenal Syndrome in Acute Heart Failure: A Systematic Review of Residual Congestion and Decongestion Response

**DOI:** 10.7759/cureus.116198

**Published:** 2026-09-13

**Authors:** Abdul Hannan, Abul Kashem Himon, Sarah Singh, Mohammad A Al Zoubi, Hashir Habib, FNU Sabeen, Rafia Saeed, Abdelrahman Shehata, Rabia Azhar, Swaira Kanwal

**Affiliations:** 1 Internal Medicine, The Rotherham NHS Foundation Trust, Rotherham, GBR; 2 Internal Medicine, University Hospital Wishaw, Wishaw, GBR; 3 Psychiatry and Behavioral Sciences, Vita-Salute San Raffaele University, Milan, ITA; 4 Internal Medicine, The University of Texas Health Science Center at Houston, Houston, USA; 5 Internal Medicine, Rawal Institute of Health Sciences, Islamabad, PAK; 6 Internal Medicine, King Edward Medical University, Lahore, PAK; 7 Internal Medicine, Gulf Medical University, Ajman, ARE; 8 Internal Medicine, Lahore Medical and Dental College, Lahore, PAK; 9 Internal Medicine, Quaid-e-Azam Medical College, Bahawalpur, PAK

**Keywords:** acute heart failure, acute kidney injury, cardiorenal syndrome, decongestion, renal dysfunction, residual congestion, worsening renal function

## Abstract

Renal dysfunction is common during hospitalization for acute heart failure and is traditionally considered a defining feature of cardiorenal syndrome type 1. However, the prognostic significance of worsening renal function may depend on the clinical context, particularly whether congestion persists or effective decongestion is achieved. This systematic review evaluated contemporary evidence regarding the relationships among renal dysfunction, residual congestion, decongestion response, and clinical outcomes in adults hospitalized with acute heart failure or cardiorenal syndrome type 1. PubMed/MEDLINE, Scopus, and Web of Science were systematically searched for studies published between January 1, 2021, and December 31, 2025. Eligible studies included randomized controlled trials, prespecified analyses of clinical trials, and post hoc analyses of trial-derived data that reported both renal and congestion-related outcomes. Five studies met the inclusion criteria. Residual congestion was consistently associated with worse clinical outcomes, whereas worsening renal function during effective decongestion did not uniformly predict adverse renal or cardiovascular events. Studies evaluating venous excess ultrasound (VExUS)-guided and bioimpedance-guided strategies suggested that objective congestion assessment may improve decongestion phenotyping, although the available evidence remains limited. Overall, the findings support a renal-congestion framework in which kidney function is interpreted alongside congestion burden, diuretic response, perfusion status, and the trajectory of renal recovery. This approach may better distinguish high-risk persistent congestion from transient renal changes occurring during successful decongestion. Future prospective studies should determine whether combined renal-congestion phenotypes improve risk stratification beyond creatinine-based definitions of cardiorenal syndrome alone.

## Introduction and background

Acute heart failure is a common cause of hospitalization and is frequently complicated by renal dysfunction during treatment. This interaction between cardiac decompensation and kidney impairment is commonly classified as cardiorenal syndrome type 1, traditionally characterized by an acute decline in renal function in the setting of cardiac illness [1]. In clinical practice, even modest increases in serum creatinine may influence treatment decisions, particularly the intensity of diuretic therapy. However, renal dysfunction in acute heart failure is biologically complex and may reflect several overlapping processes, including venous congestion, reduced renal perfusion, neurohormonal activation, diuretic exposure, pre-existing chronic kidney disease, and hemodynamic changes during decongestion [2].

Worsening renal function has historically been regarded as an adverse prognostic marker in patients hospitalized with acute heart failure. Although this association is clinically important, a creatinine-centered interpretation may be incomplete. In some patients, renal deterioration occurs alongside persistent congestion and a poor diuretic response, suggesting ongoing hemodynamic stress and unresolved volume overload [3]. In others, a transient increase in creatinine may occur during effective decongestion without necessarily indicating progressive structural kidney injury. This distinction is clinically important because undertreatment of congestion may contribute to persistent symptoms, rehospitalization, and other adverse outcomes, whereas unnecessary interruption of decongestive therapy may delay the restoration of euvolemia [4,5].

Growing evidence suggests that congestion status and the response to decongestive therapy are central to understanding the prognostic significance of renal dysfunction in acute heart failure. Residual congestion, impaired natriuretic or diuretic response, absent hemoconcentration, and persistently elevated natriuretic peptide levels may identify patients at increased risk even when renal function appears stable [6]. Conversely, successful decongestion may alter the clinical interpretation of worsening renal function, particularly when renal changes are transient and accompanied by improved volume status. Objective measures, including composite congestion scores, venous excess ultrasound (VExUS), bioimpedance analysis, natriuretic peptide trends, and markers of hemoconcentration, may therefore refine cardiorenal risk assessment beyond serum creatinine alone [7].

This systematic review aimed to evaluate contemporary evidence regarding the relationships among renal dysfunction, residual congestion, decongestion response, and clinical outcomes in adults hospitalized with acute heart failure or cardiorenal syndrome type 1. Specifically, it examined whether renal dysfunction carries independent prognostic significance or whether its clinical implications are better understood in relation to congestion burden and the adequacy of decongestion. By synthesizing studies that assessed renal outcomes alongside congestion-related variables, this review sought to support a more nuanced renal-congestion framework for interpreting cardiorenal syndrome in acute heart failure.

## Review

Materials and methods

Study Design and Reporting Framework

This systematic review evaluated the relationships among renal dysfunction, residual congestion, decongestion response, and clinical outcomes in adults hospitalized with acute heart failure. It was conducted in accordance with the Preferred Reporting Items for Systematic Reviews and Meta-Analyses (PRISMA) 2020 statement [8]. The review was not prospectively registered in PROSPERO or OSF, and no publicly accessible protocol was published. However, an internal protocol specifying the research question, eligibility criteria, information sources, outcomes, study-selection process, data-extraction approach, risk-of-bias assessment, and planned narrative synthesis was prepared before study selection and followed throughout the review. No substantive deviations from this protocol occurred. The research question was structured using the Population, Intervention or Exposure, Comparator, and Outcomes (PICO) framework. The population comprised adults hospitalized with acute heart failure, acute decompensated heart failure, or cardiorenal syndrome type 1. Interventions or exposures of interest included worsening renal function, baseline renal dysfunction, residual congestion, decongestion response, and the use of objective congestion-guided strategies. Comparators included patients without worsening renal function, those receiving usual care, those achieving successful versus incomplete decongestion, and groups stratified by renal function or congestion status. Outcomes of interest included kidney function recovery, worsening renal function, acute kidney injury, long-term decline in kidney function, residual congestion, successful decongestion, heart failure or renal failure rehospitalization, cardiovascular and all-cause mortality, and composite clinical outcomes.

Search Strategy and Data Sources

A systematic literature search was conducted using PubMed/MEDLINE, Scopus, and Web of Science for studies published between January 1, 2021, and August 1, 2026. All three databases were last searched on August 1, 2026. The strategy combined controlled vocabulary, where available, and free-text terms related to acute heart failure, renal dysfunction, cardiorenal syndrome, congestion, and decongestion. To maximize search sensitivity, clinical-outcome terms were not applied as a mandatory search component. The revised PubMed search combined the following groups of terms using Boolean operators: (“acute heart failure” OR “acute decompensated heart failure” OR “hospitalized heart failure” OR “heart failure hospitalization”) AND (“worsening renal function” OR “acute kidney injury” OR “renal dysfunction” OR “cardiorenal syndrome” OR “kidney function” OR “estimated glomerular filtration rate” OR “eGFR” OR “creatinine”) AND (“congestion” OR “residual congestion” OR “persistent congestion” OR “decongestion” OR “volume overload” OR “fluid overload” OR “diuretic response” OR “hemoconcentration” OR “bioimpedance” OR “VExUS”). Eligibility based on renal, congestion-related, and clinical outcomes was assessed during screening rather than through restrictive outcome-based search terms. The strategy was adapted for Scopus and Web of Science using equivalent title, abstract, and keyword fields. The reference lists of eligible studies and relevant review articles were also manually screened to identify additional studies.

Eligibility Criteria

Studies were eligible if they enrolled adults hospitalized with acute heart failure, acute decompensated heart failure, or cardiorenal syndrome type 1 and evaluated renal dysfunction in relation to congestion status, decongestion response, or congestion-guided management. No restriction was applied according to left ventricular ejection fraction; therefore, studies involving heart failure with reduced, mildly reduced, or preserved ejection fraction were eligible. Phenotype-specific findings were extracted when reported. Eligible designs included randomized controlled trials, prespecified analyses of randomized trials, post hoc analyses of clinical trial data, and prospective pilot interventional studies. Studies were required to report at least one renal outcome, such as worsening renal function, acute kidney injury, kidney function recovery, decline in estimated glomerular filtration rate (eGFR), or progression to advanced chronic kidney disease. They were also required to report at least one congestion-related variable, such as residual congestion, a composite congestion score, changes in natriuretic peptide levels, hemoconcentration, reversal of volume overload, venous excess ultrasound (VExUS)-guided assessment, bioimpedance-guided assessment, or clinical decongestion.

Studies were excluded if they focused exclusively on chronic stable heart failure, outpatient heart failure without recent acute decompensation, chronic kidney disease without acute heart failure, or renal outcomes unrelated to congestion or decongestive therapy. Case reports, narrative reviews, editorials, letters without original data, animal studies, pediatric studies, and studies without extractable renal or congestion-related outcomes were also excluded. Studies evaluating pharmacological or assessment-guided decongestion strategies were considered eligible if they reported relevant renal and congestion endpoints, as these studies directly informed whether renal dysfunction should be interpreted in the context of decongestion status.

Study Selection

All records identified through database searches were imported into a reference-management file, and duplicates were removed before screening. Two reviewers independently screened titles and abstracts against the predefined eligibility criteria. Full texts of potentially eligible studies were then reviewed independently by the same two reviewers. Disagreements were resolved through discussion and consensus, with involvement of a third reviewer when required. Studies were selected for primary evidence synthesis when they directly addressed the interaction between renal function, congestion status, decongestion response, or congestion-guided management in hospitalized acute heart failure or cardiorenal syndrome type 1.

Data Extraction

Data were extracted independently by two reviewers using a standardized extraction form developed before synthesis. Extracted variables included first author, year of publication, study design, source trial or cohort, population characteristics, sample size, intervention or exposure, comparator, renal variables, congestion or decongestion variables, follow-up duration, and main renal and clinical outcomes. Particular attention was given to definitions of worsening renal function, acute kidney injury, kidney function recovery, residual congestion, successful decongestion, natriuretic peptide response, hemoconcentration, and objective congestion assessment. For studies reporting adjusted associations, adjusted effect estimates and confidence intervals were extracted where available. The reviewers compared their completed forms, and disagreements were resolved through discussion and consensus. A third reviewer was consulted when consensus could not be reached.

Risk of Bias Assessment

Risk of bias was assessed using tools appropriate to study design. Randomized controlled trials and prespecified analyses of randomized controlled trials were assessed using the Cochrane Risk of Bias 2 (RoB 2) tool [9]. Non-randomized prognostic or post hoc analyses of trial-derived data were assessed using the Risk Of Bias In Non-randomized Studies of Interventions (ROBINS-I) tool [10]. The assessment considered randomization, deviations from intended interventions, missing outcome data, measurement of outcomes, selection of reported results, confounding, exposure classification, and precision of effect estimates. Overall judgments were categorized as low risk, some concerns, moderate risk, or higher concern depending on the tool used and the dominant sources of bias.

Data Synthesis

Given the clinical and methodological heterogeneity across studies, a narrative synthesis was performed rather than a meta-analysis. The included studies varied in population, congestion assessment method, renal outcome definition, intervention strategy, and follow-up duration. Therefore, findings were synthesized thematically around three domains: residual congestion as a prognostic marker, worsening renal function during decongestion, and objective methods for congestion assessment. The synthesis focused on whether renal dysfunction appeared to carry different prognostic implications depending on whether congestion persisted or improved. A phenotype-based interpretive framework was then developed to distinguish patients with persistent congestion and worsening renal function from those with successful decongestion despite transient renal deterioration.

Results

Study Selection Process

The study selection process is summarized in Figure 1. A total of 398 records were identified through searches of PubMed/MEDLINE, Scopus, and Web of Science. After removal of 42 duplicate records, 356 records were screened by title and abstract, of which 207 were excluded. A total of 149 reports were sought for retrieval. Full texts were sought through DOI resolution, journal and publisher websites, Google Scholar, institutional repositories, and direct contact with corresponding authors when contact information was available. Despite these attempts, 19 reports could not be retrieved, leaving 130 full-text reports for eligibility assessment. Following full-text assessment, 125 reports were excluded for reasons including chronic stable heart failure, outpatient heart failure without recent acute decompensation, chronic kidney disease without acute heart failure, renal outcomes unrelated to congestion or decongestive therapy, case reports, narrative reviews, editorials or letters without original data, animal studies, pediatric studies, and absence of extractable renal or congestion-related outcomes. Ultimately, five studies met the eligibility criteria and were included in the systematic review.

**Figure 1:**
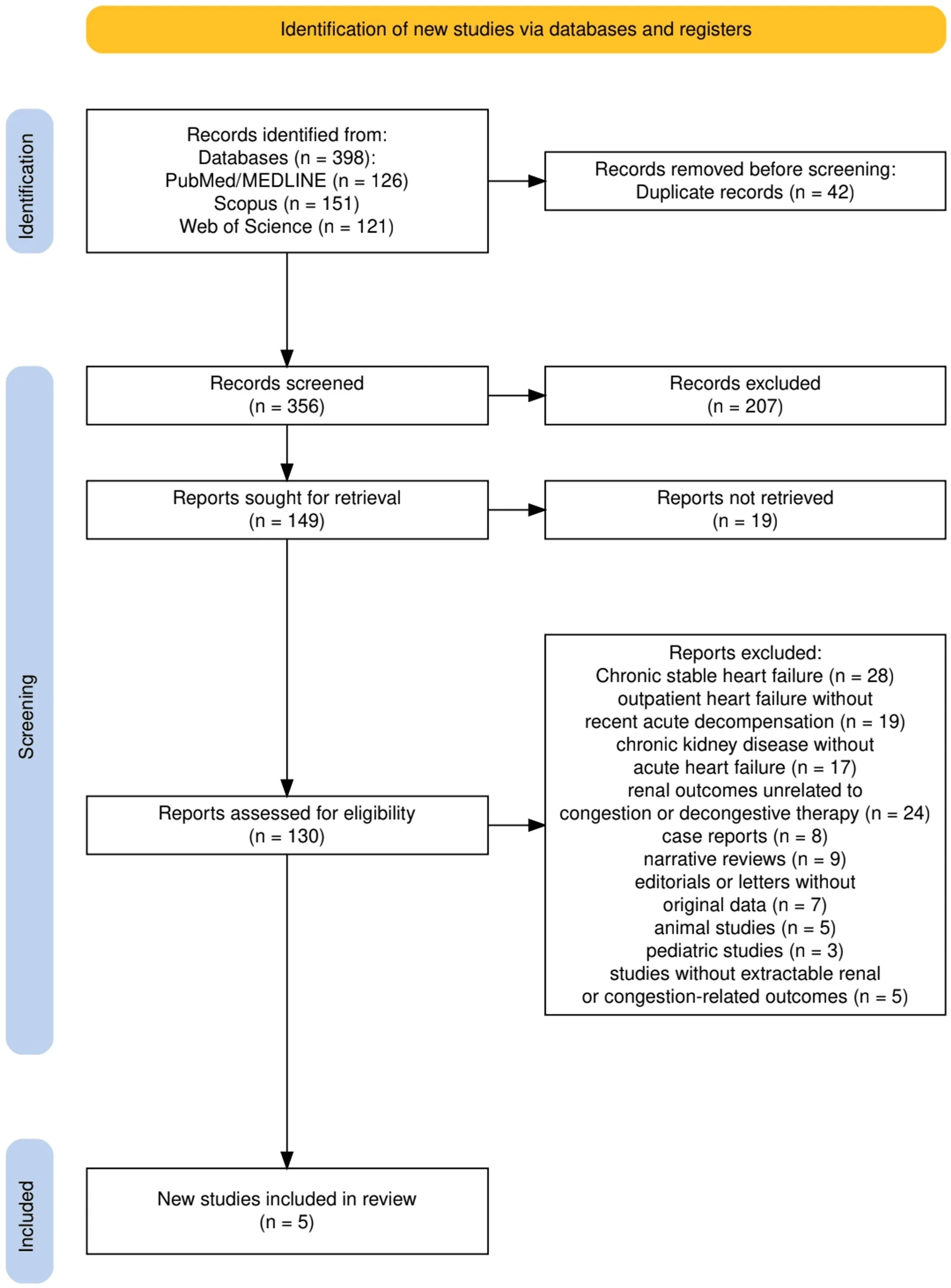
PRISMA flow diagram of the study selection process. PRISMA: Preferred Reporting Items for Systematic Reviews and Meta-Analyses

Characteristics of the Selected Studies

As summarized in Table 1, the included studies comprised randomized controlled trials, prespecified trial analyses, and post hoc analyses of trial-derived data involving adults with acute heart failure, acute decompensated heart failure, or cardiorenal syndrome type 1. The study populations ranged from small pilot cohorts to large multicentre trial cohorts, with sample sizes from 48 to 5,900 participants. Across the included evidence, renal variables included worsening renal function, acute kidney injury, kidney function recovery, eGFR decline, and progression to advanced chronic kidney disease, while congestion-related variables included residual congestion scores, natriuretic peptide changes, hemoconcentration, VExUS-guided assessment, bioimpedance analysis, and clinical decongestion. Together, these studies provided a focused evidence base for evaluating whether renal dysfunction in acute heart failure is better interpreted in relation to congestion status and decongestion response rather than as an isolated prognostic marker.

**Table 1:** Characteristics and principal findings of the included studies. ADVOR, Acetazolamide in Decompensated Heart Failure with Volume Overload; AKI, acute kidney injury; BIA, bioelectrical impedance analysis; BNP, B-type natriuretic peptide; CA-125, cancer antigen 125; CCS, composite congestion score; CI, confidence interval; CKD, chronic kidney disease; CRS1, cardiorenal syndrome type 1; eGFR, estimated glomerular filtration rate; EVEREST, Efficacy of Vasopressin Antagonism in Heart Failure: Outcome Study With Tolvaptan; HF, heart failure; HR, hazard ratio; JVP, jugular venous pressure; NT-proBNP, N-terminal pro-B-type natriuretic peptide; OR, odds ratio; RELAX-AHF-2, Relaxin in Acute Heart Failure 2; VExUS, venous excess ultrasound; WRF, worsening renal function.

Study	Design/Source	Population	Main Exposure/Intervention	Renal Variable	Congestion/Decongestion Variable	Key Findings
Islas-Rodríguez et al., 2024 [11]	Randomized controlled trial	Patients with CRS1; n = 140	VExUS-guided decongestion versus usual clinical evaluation	CRS1 defined as creatinine increase ≥0.3 mg/dL; kidney function recovery was the primary endpoint	Physical examination and changes in BNP and CA-125	• No significant difference in kidney function recovery. • Greater decongestion with VExUS guidance (OR 2.6; 95% CI 1.9–3.0; p = 0.01). • BNP reduction >30% was more frequent (OR 2.4; 95% CI 1.3–4.1; p = 0.01).• Similar 90-day survival and recongestion.
McCallum et al., 2022 [12]	Post hoc analysis of EVEREST trial data	Hospitalized patients with acute HF and ≥2 kidney-function measurements; n = 3,500	In-hospital rate of volume-overload reversal and hemoconcentration	Incident CKD G4–G5 (new eGFR <30 mL/min/1.73 m²) and eGFR decline >40%	Changes in BNP, NT-proBNP, clinical congestion score, hematocrit, albumin, and total protein	• Faster decongestion was associated with better long-term kidney outcomes. • Each 6% faster weekly BNP decline reduced the risks of CKD G4–G5 and >40% eGFR decline (HR 0.68 for both). • Faster hematocrit rise reduced these risks (HR 0.73 and 0.82, respectively).
Venegas-Rodríguez et al., 2023 [13]	Single-centre, single-blind randomized controlled pilot study	Overweight or obese patients admitted with acute HF; n = 48	BIA-guided management versus standard care	Severe AKI defined as creatinine increase >0.5 mg/dL; kidney function assessed during hospitalization and at 90 days	BIA-guided volume assessment and NT-proBNP measurements	• Severe AKI was numerically lower with BIA guidance (16.7% vs 41.4%; p = 0.057). • NT-proBNP <1,000 pg/mL at 90 days was more frequent with BIA guidance (58.8% vs 25.0%; p = 0.049). • No difference in 90-day adverse outcomes.
Pagnesi et al., 2025 [14]	Secondary analysis of RELAX-AHF-2 trial data	Hospitalized patients with acute HF who remained admitted at day 5; n = 5,900	Residual congestion at day 5	WRF at day 5	CCS based on orthopnea, peripheral edema, and increased JVP; residual congestion defined as CCS ≥1 and severe residual congestion as CCS ≥3	• Residual congestion increased the 180-day risk of cardiovascular death or HF/renal failure rehospitalization. • CCS ≥1: adjusted HR 1.32 (95% CI 1.15–1.51; p < 0.001). • CCS ≥3: adjusted HR 1.62 (95% CI 1.39–1.88; p < 0.001).
Meekers et al., 2023 [15]	Prespecified analysis of the ADVOR randomized controlled trial	Patients with acute decompensated HF; n = 519	Intravenous acetazolamide plus loop diuretics versus placebo plus loop diuretics	Baseline eGFR; median 40 mL/min/1.73 m²; WRF defined as creatinine increase ≥0.3 mg/dL	Decongestion, diuresis, and natriuresis across baseline eGFR levels	• Acetazolamide improved decongestion across all eGFR levels. • Natriuresis and diuresis increased, particularly at lower eGFR. • WRF was more frequent (40.5% vs 18.9%; p < 0.001) but did not increase HF hospitalization or mortality. • Decongestion predicted better outcomes regardless of WRF.

Risk of Bias Assessment

As shown in Table 2, risk of bias was assessed using tools selected a priori according to the estimand evaluated in each analysis. The revised Cochrane Risk of Bias tool for randomized trials (RoB 2) was applied when the estimand concerned the effect of a randomized intervention. ROBINS-I was retained for post hoc analyses in which the estimands were comparative associations between predefined, non-randomized exposure states, such as residual versus resolved congestion or faster versus slower decongestion, and subsequent clinical outcomes. These analyses were not treated as randomized merely because they used data from randomized trials. ROBINS-I was considered suitable for evaluating the principal threats to validity in these exposure-based comparisons, particularly confounding, participant selection, exposure classification, missing data, outcome measurement, and selective reporting. Although Quality In Prognosis Studies (QUIPS)is commonly used for prognostic-factor studies, the included analyses primarily estimated outcome differences between defined exposure groups rather than developing or validating prognostic models. We therefore retained the prespecified ROBINS-I framework to maintain methodological consistency while interpreting its findings as assessments of non-randomized associations rather than causal treatment effects. Table 2 presents the overall judgments and identifies the domain-level considerations that contributed to each judgment. The post hoc analyses were judged to have a moderate risk of bias, driven mainly by potential residual confounding because congestion status and the rate of decongestion were not randomized. Accordingly, all studies were retained in the narrative synthesis, but causal interpretations were avoided.

**Table 2:** Risk-of-bias assessment of the included studies. eGFR, estimated glomerular filtration rate; RCT, randomized controlled trial; RoB 2, Risk of Bias 2 tool; ROBINS-I, Risk Of Bias In Non-randomized Studies of Interventions; VExUS, venous excess ultrasound

Study	Tool Applied	Overall Risk of Bias	Rationale
Islas-Rodríguez et al., 2024 [11]	RoB 2	Some concerns	Randomized design supports internal validity, but VExUS-guided treatment could not be fully blinded, and decongestion assessment included physical examination, which may introduce assessment bias.
McCallum et al., 2022 [12]	ROBINS-I	Moderate	Post hoc observational analysis of trial data. Faster decongestion was not randomized, so residual confounding by illness severity, treatment response, and baseline kidney risk is possible.
Venegas-Rodríguez et al., 2023 [13]	RoB 2	Some concerns	Randomized pilot study, but small sample size, single-centre design, and limited power increase imprecision and risk of unstable effect estimates.
Pagnesi et al., 2025 [14]	ROBINS-I	Moderate	Residual congestion at day 5 was analyzed as a prognostic exposure, not as a randomized intervention. Multivariable adjustment reduces but does not eliminate confounding.
Meekers et al., 2023 [15]	RoB 2	Low to some concerns	Pre-specified RCT analysis with objective renal outcomes. Some caution remains for subgroup interpretation across baseline eGFR strata.

Discussion

Principal Findings

This review suggests that renal dysfunction in acute heart failure should be interpreted within the broader context of congestion burden and response to decongestive therapy. Across the included studies, persistent or residual congestion was a more consistent marker of adverse prognosis than renal dysfunction considered in isolation. Pagnesi et al. [14] demonstrated that residual congestion during hospitalization was independently associated with worse clinical outcomes. Meanwhile, findings from Meekers et al. [15] and McCallum et al. [12] indicated that effective decongestion may occur despite transient worsening of renal function, without necessarily leading to adverse renal or clinical outcomes. Collectively, these findings support a shift from a creatinine-centered interpretation of cardiorenal syndrome toward an integrated renal-congestion framework in which the prognostic significance of kidney dysfunction depends partly on whether congestion is relieved or persists.

Cardiorenal Syndrome as a Congestion-Dependent Phenotype

Traditional definitions of cardiorenal syndrome type 1 rely heavily on an acute increase in serum creatinine, commonly defined using a threshold of at least 0.3 mg/dL. Although clinically useful, this definition may not adequately capture the heterogeneity of renal dysfunction during the treatment of acute heart failure. An increase in creatinine during successful decongestion may represent a biologically and prognostically distinct state from a similar increase occurring alongside persistent venous congestion, poor diuretic response, and residual volume overload [16]. The findings of Meekers et al. [15] are particularly relevant in this context: worsening renal function occurred more frequently with acetazolamide, yet it was not associated with poorer clinical outcomes, while successful decongestion was associated with a more favorable prognosis. These findings support a more nuanced interpretation in which the prognostic significance of worsening renal function depends less on the creatinine increase alone and more on the clinical context in which it occurs.

Residual Congestion as a Prognostic Marker

Residual congestion emerged as an important marker of postdischarge risk in patients hospitalized with acute heart failure. In the analysis by Pagnesi et al. [14], residual congestion at day 5 was common and independently associated with a higher 180-day risk of cardiovascular death, rehospitalization for heart failure, or rehospitalization for renal failure. This finding suggests that incomplete decongestion represents more than persistent symptom burden; it may reflect ongoing hemodynamic stress, venous hypertension, impaired diuretic responsiveness, and unresolved organ congestion. Importantly, stable renal function should not be considered reassuring when congestion persists. Patients with stable creatinine but residual congestion may remain at substantial clinical risk, and renal stability in the absence of adequate decongestion may create a false impression of therapeutic success [17].

Reinterpreting Worsening Renal Function During Decongestion

The available evidence challenges the assumption that worsening renal function during decongestive therapy invariably represents harmful kidney injury. In the randomized ADVOR analysis by Meekers et al. [15], acetazolamide improved decongestion across different levels of baseline renal function but increased the incidence of worsening renal function. Nevertheless, worsening renal function was not associated with higher rates of heart failure hospitalization or mortality during the three-month follow-up, and serum creatinine levels did not differ between the treatment groups at three months [15]. In the post hoc EVEREST analysis by McCallum et al. [12], faster reversal of volume overload and greater hemoconcentration were associated with lower risks of adverse kidney outcomes over a median follow-up of 10 months. Because the latter findings were based on non-randomized exposure comparisons, they should not be interpreted as demonstrating a causal protective effect of faster decongestion [12]. Collectively, these findings suggest that some increases in creatinine occurring alongside effective decongestion may represent transient functional or hemodynamic changes rather than progressive structural kidney injury [12,15]. However, given the small and heterogeneous evidence base, these findings should be considered interpretive rather than formal treatment guidance. They support considering congestion relief, perfusion status, blood pressure, urine output, electrolyte stability, and subsequent renal recovery when interpreting worsening renal function, but they do not establish a universal recommendation regarding the continuation or discontinuation of decongestive therapy [18].

Venous Congestion as the Mechanistic Bridge

The relationship between acute heart failure and renal dysfunction is often explained through reduced forward flow, but this mechanism alone does not fully account for the observed clinical pattern. Venous congestion provides an important mechanistic bridge between cardiac decompensation, renal impairment, and treatment response. Elevated central and renal venous pressures can reduce the transrenal perfusion gradient, increase renal interstitial pressure, impair tubular function, and contribute to diuretic resistance [19]. In this context, the kidney in acute heart failure may be injured not only by hypoperfusion, but also by pressure from behind. This pathophysiological model helps explain why effective decongestion may improve renal trajectory even when serum creatinine rises transiently during treatment. It also supports a more integrated interpretation of cardiorenal syndrome, in which renal dysfunction is viewed as a dynamic consequence of both arterial perfusion and venous congestion rather than as an isolated biochemical event [2].

Objective Congestion Assessment Beyond Physical Examination

If congestion status modifies the prognostic meaning of renal dysfunction, then accurate assessment of congestion becomes central to both clinical care and research in cardiorenal syndrome. The included studies used several complementary approaches, including physical examination, composite congestion scoring, B-type natriuretic peptide (BNP) or N-terminal pro-B-type natriuretic peptide (NT-proBNP) trends, hemoconcentration markers, VExUS, and bioimpedance analysis. Islas-Rodríguez et al. [11] showed that VExUS-guided decongestion improved the odds of achieving decongestion and BNP reduction, although it did not significantly improve kidney function recovery. Similarly, Venegas-Rodríguez et al. [13] suggested that bioimpedance-guided management may improve congestion-related biomarker response and may reduce severe AKI, although the study was small and exploratory. These findings suggest that objective tools may improve congestion phenotyping, particularly when physical examination is limited or discordant with biomarkers. However, VExUS and bioimpedance should currently be viewed as adjunctive methods rather than definitive replacements for clinical assessment, pending larger studies that test whether tool-guided decongestion improves hard renal and cardiovascular outcomes [7].

A Practical Four-Phenotype Framework

A hypothesis-generating implication of this synthesis is that patients with acute heart failure may be considered through a combined renal-congestion framework rather than through renal function alone [5,6]. Within this conceptual framework, four potential phenotypes can be proposed: a congested-worsening renal function (WRF) phenotype, in which creatinine rises while congestion persists; a decongested-WRF phenotype, in which creatinine rises despite improvement in congestion; a congested-stable renal phenotype, in which creatinine remains stable but congestion persists; and a decongested-stable renal phenotype, in which renal function remains stable while congestion improves [20]. This framework may help separate the renal signal from the congestion signal and offers a possible explanation for the inconsistent prognostic significance of worsening renal function across studies. The same increase in creatinine may have different implications depending on whether it occurs alongside unresolved venous congestion and poor diuretic response or during effective decongestion. However, these four phenotypes were not prospectively evaluated as a unified classification in the included studies. The framework should therefore be regarded as conceptual and hypothesis-generating rather than as a validated clinical classification or decision-making tool.

Limitations

This review has several important limitations. First, only five studies met the eligibility criteria, limiting the breadth and certainty of the evidence. The included studies also demonstrated substantial clinical and methodological heterogeneity in their populations, study designs, sample sizes, definitions of worsening renal function and decongestion, assessment methods, interventions or exposures, and follow-up durations. The synthesis combined randomized intervention effects with findings from post hoc and prognostic analyses of trial-derived data. The latter remain susceptible to residual confounding and should be interpreted as associations rather than causal effects. The restricted publication period from January 1, 2021, to August 1, 2026, was selected to capture contemporary evidence but may have excluded relevant earlier studies. In addition, 19 reports could not be retrieved despite multiple retrieval attempts. Because their eligibility and findings could not be assessed, non-retrieval may have introduced selection bias. Finally, the heterogeneity of the included studies prevented quantitative pooling and precluded estimation of a summary effect or formal assessment of between-study statistical heterogeneity. These limitations restrict the certainty and generalizability of the conclusions, which should be regarded as interpretive and hypothesis-generating.

Clinical and Research Implications

These findings support a more cautious and contextual approach to renal dysfunction during acute heart failure treatment. Clinically, a rise in creatinine should not be interpreted in isolation, nor should mild worsening renal function automatically lead to premature interruption of decongestive therapy when congestion persists. Conversely, stable renal function should not be viewed as evidence of successful treatment if residual congestion remains. Discharge assessment should therefore integrate both renal function and congestion status, using clinical examination, biomarkers, hemoconcentration, urine output, and objective congestion tools where appropriate [18]. From a research perspective, future studies should prospectively test combined renal-congestion phenotypes and determine whether they predict mortality, rehospitalization, renal recovery, and long-term kidney decline better than creatinine-based definitions of cardiorenal syndrome alone. The next step in cardiorenal research may not simply be earlier identification of renal injury, but clearer identification of which renal changes matter in the context of congestion response [20].

## Conclusions

This review supports moving beyond a solely creatinine-based interpretation of cardiorenal syndrome in acute heart failure. The available evidence suggests that the prognostic significance of renal dysfunction is strongly influenced by congestion status and the adequacy of decongestion. Residual or persistent congestion appears to characterize a higher-risk clinical state, whereas worsening renal function during effective decongestion may, in selected patients, reflect a transient functional response rather than progressive kidney injury. Accordingly, kidney function should be evaluated alongside congestion burden, diuretic response, perfusion status, and the trajectory of renal recovery. Future prospective studies should determine whether combined renal-congestion phenotypes improve risk stratification and treatment guidance beyond conventional creatinine-based definitions of cardiorenal syndrome.
